# A quantitative comparison of bone resection margin distances in virtual surgical planning versus histopathology: a prospective study

**DOI:** 10.1097/JS9.0000000000000780

**Published:** 2023-09-21

**Authors:** Jane J. Pu, Anthony W. I. Lo, May C. M. Wong, Wing-Shan Choi, Grace Ho, Wei-Fa Yang, Yu-Xiong Su

**Affiliations:** a Division of Oral and Maxillofacial Surgery; b Division of Applied Oral Sciences and Community Dental Care, Faculty of Dentistry, The University of Hong Kong; c Department of Anatomical Pathology; d Department of Radiology, Queen Mary Hospital, Hong Kong Special Administrative Region, People’s Republic of China

**Keywords:** 3D printing, computer-assisted surgery, jaw reconstruction, surgery margin, virtual surgical planning

## Abstract

**Background:**

Positive bone margins have been shown to be associated with worse locoregional control and survival performance in oral oncology patients. With the application of computer-assisted surgery and patient-specific surgical guides, the authors can accurately execute the preoperative osteotomy plan. However, how well the authors can predict the margin distance in the final histopathology with a preoperative computed tomography (CT) scan, the factors associated with it, and how much leeway CT should spare when designing the osteotomy planes during virtual surgical planning (VSP) remain to be investigated.

**Materials and methods:**

Patients from January 2021 to December 2022 with benign or malignant jaw tumors and with signs of bone marrow involvement in the preoperative CT scan in our center were prospectively recruited to the study. VSP and measurement of the closest margin distance in the CT scan were performed by the single team of surgeons. The resection specimen was processed, and the margin distances were measured by a dedicated senior pathologist with the knowledge of orientation of the osteotomy planes.

**Results:**

A total of 35 patients were recruited, with 21 malignant and 14 benign cases. Sixty-eight bone margins were quantitatively analyzed. No significant difference in margin distances measured from the CT scan and final histopathology was detected (*P*=0.19), and there was a strong correlation between the two (r_s_=0.74, *P*<0.01). A considerable amount of variance was detected in the level of discrepancy between margin distances measured in the CT scan and final histopathology (overall SD=6.26 mm, malignancy SD=7.44 mm, benign SD=4.40 mm). No significant correlation existed between the two margin distances when only maxilla tumor margins were assessed (*P*=0.16).

**Conclusion:**

The bone margin distance in VSP is reliably correlated to the final pathological margin distance. A leeway distance of 15mm and 9mm should be considered when designing the osteotomy planes for malignancy and benign cases, respectively. Extra attention should be paid to maxilla cases when predetermining the osteotomy planes during VSP.

## Introduction

Highlights

- Bone margin distance in virtual surgical planning based on computed tomography scan can reliably predict the final pathological margin distance.
- A leeway distance of 15 mm and 9 mm should be considered when designing the osteotomy planes for malignancy and benign cases, respectively.
- Extra attention needs to be paid to maxillary tumors and sarcoma cases during virtual surgical planning.

Inadequate resection margins have been shown to be associated with worse locoregional control and survival performance in oral oncology patients^1,2^. Compared to the soft tissue margins, positive bone margins might have an even bigger survival impact^3^. However, over-resection in the head and neck area also can lead to the loss of vital structures and difficult reconstruction. Precision surgery aims to minimize the destruction without compromising oncological safety.

Regardless of the great importance of accurate bone margins, several challenges exist. Preoperatively, it is often difficult to precisely locate the position of the tumor margin in bone even with multiple imaging techniques^4^. During the operation, there is so far no reliable way to check the bone margin using a frozen section (FS). Technologies like cytologic assessment or curettage of cancellous bone can help detect malignant cells in the resection margin, but the reliability is still less than ideal^5,6^. The information on bone margin in the pathology report is only available after a long period of decalcification^7^. If inadequate bone margin is reported in the final pathology, further resection requires reoperation and often revised reconstruction. A delay or upgrade in adjuvant treatment is often necessary in such cases.

To avoid resection with an inadequate margin, the osteotomy plane needs to be properly designed from the preoperative imaging and accurately executed during the operation. Empirically, a margin of 0.5–1 cm from suspicious bone was adopted for oral squamous cell carcinoma^4^. A margin of more than 15 mm for better survival performance was also proposed^3^. However, how well these numbers can help us design an oncologically-safe osteotomy plane without over-resection remains unknown.

In the past, it was often difficult to compare the margin distance measured in imaging with the ʻgolden standardʼ measurement from the final histopathology specimen. With free-hand surgery, surgeons could neither measure the distance from the osteotomy plane to the bone invasion front nor compare the clinical resection margin distance with the radiological or pathological distance. Computer-assisted surgery has transformed jaw resection and reconstruction surgeries in the past decades^8^. 3D-printed patient-specific resection guides could precisely locate the osteotomy plane according to virtual surgical planning (VSP) based on imaging^7,9,10^, thus making the comparison between the ʻplanned resection marginʼ and ʻfinal pathological marginʼ possible. This prospective study was designed to quantitatively evaluate the reliability of margin distances measured in the preoperative surgical plan in predicting margin status in the final histopathology and associated factors, and to provide guidance on the design of osteotomy planes during VSP based on preoperative imaging.

## Methods

### Patients’ data collection

This study was a single-centered prospective cohort study. It was approved by the Institutional Review Board (UW 15-315). Patients with malignant or benign tumors at the maxilla or mandible with evidence of marrow involvement in a preoperative computed tomography (CT) scan who were planned to undergo primary surgical resection and computer-assisted bone flap reconstruction in our center, from 2021 January to 2022 December were invited to participate in the study. Written informed consents were obtained from all the participants. The work has been reported in line with the strengthening the reporting of cohort, cross-sectional, and case–control studies in surgery (STROCSS) criteria^11^.

#### Inclusion criteria

1. Age greater than or equal to 18 years, both sexes;
2. Provided the signed and dated informed consent form;
3. Diagnosed with maxillofacial benign or malignant tumors with obvious marrow involvement that were indicated for computer-assisted jaw resection and reconstruction surgeries.

#### Exclusion criteria

1. Patients who are pregnant;
2. Patients who have medically compromised conditions and cannot tolerate surgery;
3. Patients who had neoadjuvant therapy before the surgery.

### VSP and surgery

**Figure 1:**
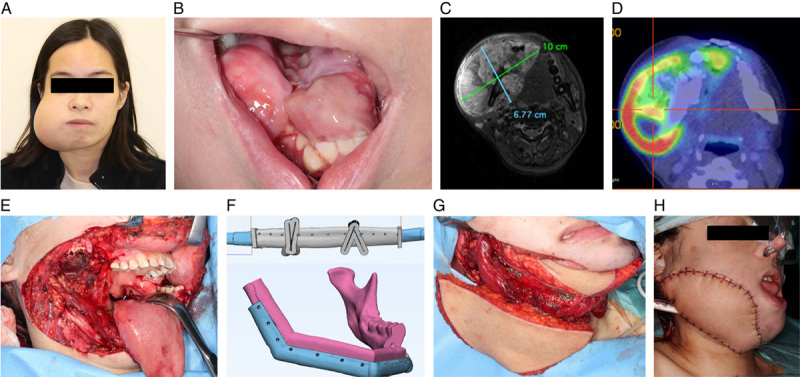
Chondroblastic osteosarcoma of mandible in a 39-year-old Chinese female. (A) Extraoral view; (B) Intraoral view; (C) Axial fat-saturated T2-weighted MRI image of the tumor; (D) PETCT scan of the tumor; (E) Defect after tumor resection and right partial mandibulectomy; (F) Virtual surgical plan of the fibula free flap reconstruction with osteotomy and positioning guides; (G) After inset of the fibula and antero-lateral thigh (ALT) free flaps; (H) After wound closure, ALT flap for extraoral skin defect and fibula free flap skin paddle for intraoral mucosal defect.

**Figure 2:**
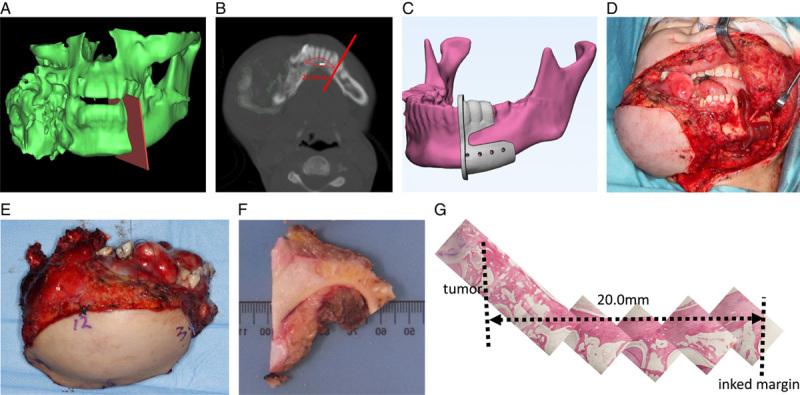
Chondroblastic osteosarcoma of mandible in a 39-year-old Chinese female. (A) Virtual model built based on CT scan of the tumor; (B) The closest distance from tumor margin in bone was measured to the resection plane in bone window of CT scan; (C) Osteotomy guide design with rest on tooth surfaces; (D) Tumor resection according to the osteotomy guide intraoperatively; (E) Tumor specimen sent for histopathology; (F) Sectioning of specimen perpendicular to cutting plane into thin slices of bone; (G) After decalcification, trimming and embedding, the margin distance from the tumor front to the inked resection margin was measured under light microscopy on the H&E section.

The detailed procedures of computer-assisted tumor resection and jaw reconstruction were described in our previous publications^12^. Briefly, a high-resolution CT scan with and without contrast was performed for patients diagnosed with benign or malignant jaw tumors (tube voltage of 140 KVp, table speed of 40 mm/rotation, gantry tilt of 0°, and slice thickness of 0.625 mm). Both soft tissue and bone windows were used to assess the extent of tumors in the jaw. Cases with obvious marrow involvement were recruited for this study. MRI and PETCT were acquired for the selected cases. An intraoral optical scan was performed to capture the soft tissue extension of the tumor and details of the dentition to facilitate rehabilitation. A composite virtual model was built with ProPlan CMF 2.0 software (Materialize). Virtual resection plans were made by the same senior surgeon with adequate margins assessed from both the clinical examination and imaging, with consideration of anatomical landmarks and reconstruction plans [Fig. 1]. The closest distance from the tumor margin in the bone was measured to the resection plane in the bone window of the CT scan. The patient-specific resection guides were designed with rest on tooth surfaces to guide the accurate mandibulectomy or maxillectomy during the surgery. All resections were performed according to the patient-specific cutting guides [Fig. 2].

### Pathological assessment

The surgical specimens were processed in a standard hospital-based Anatomical Pathology Laboratory, by a single dedicated pathologist, with knowledge of the images of the VSP and osteotomy planes prior to grossing of the specimen to guide the sectioning of bone. The exact number of margin distance measured from the VSP was blinded to the pathologist. A dedicated hospital chain saw device (EXAKT 312 Pathology Saw) was used for sectioning the bone. In brief, bones were sectioned perpendicular to the cut surfaces at the resection margins towards the tumor coarsely at several levels to identify the closest distances. Thin sections of 3–5 mm thick were taken and decalcified, before further trimming off and embedding. Where possible, the tumor and the resection margins were arranged to be embedded in one block to facilitate measurement under light microscopy on the H&E sections [Fig. 2]. The pathology report was issued according to the College of American Pathologists Cancer Reporting Protocol using American Joint Committee on Cancer Staging System 8th edition^13,14^.

### Primary outcome: reliability of margin prediction in VSP

The closest bony margin distance measured in the histopathology (pathology margin) was considered as the gold standard, which was compared and correlated with the closest distance measured from the CT image in the preoperative plan (CT margin).

### Secondary outcome: factors affecting the reliability of margin prediction

Possible factors affecting the reliability of bony margin prediction, such as type (benign vs. malignancy) and location of tumors (maxilla vs. mandible), were assessed.

### Statistical analyses

Statistical analyses were performed using IBM SPSS Statistics Version 25. The normality of the data was tested. A Wilcoxon signed-rank test was used to identify if there was any significant difference between the bony margin distances measured in the CT scan and the final pathology as most data did not follow normal distributions. Bland–Altman analysis was applied to test the level of agreement between the margin distances measured in CT and histopathology and provide guidance for determining the osteotomy plane with preoperative imaging. A Spearman correlation test was performed to identify the level of correlation between the two margins. A *P*-value less than 0.05 was considered statistically significant.

## Results

### Patient demographics

A total of 35 patients were prospectively recruited, with 21 malignancy cases and 14 benign jaw tumors. Patient demographic data and disease characteristics are shown in Table 1. The average age of the patients on diagnosis was 54.9 years. Two-thirds of the patients were male. Most of the tumors were in the mandible. Malignancy occupied 60% of the cases with the majority being squamous cell carcinoma. The rest 40% of the cases were benign jaw tumors with the most common one being ameloblastoma. The average time from the date of imaging to the operation was less than 3 weeks for malignancy cases (18.8±7.0 days) and more than 2 months for benign cases (68.1±23.5 days).

**Table 1:** Patient demographic data and disease characteristics.

Number of Patients	35
Bone margins	68
Negative in pathology	67 (98.5%)
Positive in pathology	1 (1.5%)
Sex	
Male	22 (62.9%)
Female	13 (37.1%)
Smoking	
Yes	3 (8.6%)
No	32 (91.4%)
Drinking	
Yes	1 (2.9%)
No	34 (97.1%)
Location	
Maxilla	5 (14.3%)
Mandible	30 (85.7%)
Pathology	
Malignancy	21 (60.0%)
SCC	15
Sarcoma	3
Ca ex PA	2
Adenocarcinoma	1
Benign	14 (40.0%)
Ameloblastoma	10
Ossifying fibroma	2
Odontogenic fibroma	1
Myxoma	1

Sixty-eight corresponding bone margins in CT scans and pathology reports were available for analysis. Only one positive bone margin was identified in the final pathology. FS for soft tissue margins were sent for all malignancy cases. Bone marrow samples were sent only for selected cases when in doubt. No revision of the bone margin was required during surgery due to the positive intraoperative FS report. Two positive soft tissue margins were identified in one case of squamous cell carcinoma. Details of patient demographics are presented in Table 1.

### Primary outcome: reliability of margin prediction in VSP

**Figure 3:**
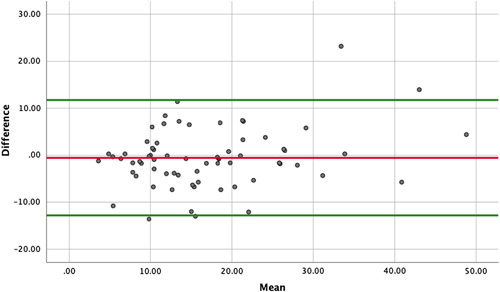
Bland-Altman plot of difference in margin distances measured in CT and pathology against the mean of two measurements. Red line: mean difference between the bone and CT margin distances. Green line: the 95% limits of agreement (the interval of 1.96 SD of the measurement differences on either side of the mean difference).

A total of 68 bony margins were available for analysis with corresponding distance measured from both the VSP and the final histopathology specimen. No significant difference was detected between the margin distances measured from VSP (17.24±8.95mm) and the final pathology report (16.71±10.54 mm) (Wilcoxon signed-ranks test, *P*=0.190). A strong correlation was detected between the margin distances measured (r_s_=0.74, *P*<0.001). The mean difference between margin distances measured from histopathology and VSP was 0.54 mm (SD=6.26 mm). The results are shown in Table 2, and the Bland–Altman plot is shown in Figure 3. The interval which included 95% of the difference was 12.28 mm on either side of the mean difference (−12.82 mm, 11.74 mm).

**Table 2:** Differences and correlations between the margin distances measured in VSP and histopathology.

	Bony margins	Mean (SD) mm	Median (IQR)	p^a^	r	p^b^
Total	VSP	17.24 (8.95)	17.00 (9.83, 21.95)	0.190	0.74	<0.001^*^
68 margins	Pathology	16.71 (10.54)	14.50 (9.18, 21.75)			
	Difference	−0.54 (6.26)				
Malignancy	VSP	21.92 (8.83)	20.80 (17.35, 26.30)	0.230	0.74	<0.001^*^
38 margins	Pathology	20.91 (11.87)	18.50 (11.75, 27.00)			
	Difference	−1.02 (7.44)				
Benign	VSP	11.31 (4.50)	9.85 (7.98, 15.20)	0.491	0.52	0.003^*^
30 margins	Pathology	11.39 (4.95)	11.00 (7.00, 14.50)			
	Difference	0.07 (4.40)				
Maxilla	VSP	8.98 (2.62)	8.60 (6.70, 10.75)	0.833	0.66	0.053
9 margins	Pathology	9.36 (4.45)	7.00 (6.00, 12.50)			
	Difference	0.38 (3.93)				
Mandible	VSP	18.50 (8.90)	18.30 (10.80, 22.30)	0.184	0.73	<0.001^*^
59 margins	Pathology	17.83 (10.77)	16.00 (11.00, 25.00)			
	Difference	−0.67 (6.57)				

* Significance with *P*<0.05.

a Significance of Wilcoxon signed-ranks test between the margin distances in VSP and histopathology.

b Significance of Spearman’s rank correlation.

IQR, interquartile range; VSP, virtual surgical plan.

### Secondary outcome: factors affecting the reliability of margin prediction

#### Malignancy versus benign

When we separated malignancy and benign cases, 38 and 30 margins were available for analysis, respectively. As shown in Table 2, no significant difference was detected between the margin distances measured in VSP and in the final pathology report for both benign and malignant cases. A strong correlation was detected between the two margin distances in the malignancy case group (r_s_=0.74, *P*<0.001). While only a moderate correlation was detected in the benign case group (r_s_=0.52, *P*=0.003). However, accordingly to the Bland–Altman analysis, benign cases showed less variation in the discrepancy between the margin distances measured from pathology and CT scan than malignancy cases (SD=4.40 mm vs. 7.44 mm). When the interval which included 95% of the difference was considered, a leeway distance of 8.80 mm and 14.88 mm should be included for benign and malignancy cases, respectively.

#### Maxilla versus mandible

When maxilla cases were assessed, no significant correlation between the two margins was detected, showing a poor predictability of margin distance in maxilla (*P*=0.053). While a strong correlation was detected for the margins measured in the mandible (r_s_=0.73, *P*<0.001).

#### Soft tissue margin versus bony margin

For malignancy cases, the soft tissue margin (12.58±8.89 mm) was significantly closer than the bony tissue margin (20.91±11.87 mm) (Wilcoxon signed-ranks test, *P*<0.001). No significant correlation was detected between soft tissue and bony tissue margins (r_s_=0.15, *P*=0.355).

## Discussion

To the best of our knowledge, this is the first prospective study designed to quantitatively verify the reliability of VSP in predicting the final pathological margin distance in jaw surgery. For benign and malignant cases with marrow involvement in preoperative imaging, margins measured during VSP based on CT scan could be used to predict the final pathological margin distance. However, attention must be paid when applied to maxillary tumors and sarcoma cases. A leeway distance of 13 mm in general, 15 mm for malignancy cases, and 9 mm for benign cases should be considered when designing the osteotomy plane during VSP.

Bony margin distance is not routinely reported in most of the centers worldwide. Getting longitudinal margins in the bony specimen is often tedious. Chain saw device is not always readily available. Fragmentation of the specimen might happen during the longitudinal sectioning of the bony specimen. During a busy routine service day, a pathologist might choose to report a shaved margin, and rely on the gross estimation of the distances between the tumor and the bone. That makes it more important to estimate the bony margin distance based on a CT scan, when the true bony margin distance in histopathology is not always available. However, in the free-hand surgery era, the CT imaging could not be correlated to the osteotomy plane during surgery, which made the estimation of bone resection margin distance impossible. Computer-assisted surgery and VSP provide an accurate tool to quantify the bone resection margin distance during surgery, making the comparison of the planned bone margin distance and the final pathological margin distance possible. Our result showed that bone margin distance in VSP could be considered as a surrogate outcome of pathological bone margin distance.

This study proved the reliability of determining the bone resection margin based on a CT scan during VSP. However, a leeway distance of 15 mm and 9 mm should be considered for malignancy and benign cases, respectively. Different clinical and imaging modalities come with different sensitivity and specificity in detecting the bony margins. CT provides higher specificity than MRI in identifying bone invasion^15^. However, in the past, people considered that CT scan underestimated the margin of invasive disease by about 5 mm^16^. This was challenged later and several authors proposed the resection with at least 10 mm of margin from the suspected bone involvement^17–19^. In the study by Singh *et al*., 10% of patients had microscopic spread along the bone with a mean distance of 0.71 cm from the macroscopic margin^3^. Several factors affecting the assessment of bony margin in imaging were proposed by Lubek *et al*., such as previous dental extraction or surgical curettage, periodontal bone loss, and active inflammation leading to false positive results in MRI/PET. Thus, high-resolution CT with fine sliced images (0.75 mm) was recommended^4^. In the current study, only cases with obvious marrow involvement were included. Fine sliced bone window CT was proved to be accurate in predicting the extension of disease when marrow space involvement was evident. However, both overestimation and underestimation from the CT scan existed. When designing the osteotomy margin, other factors including the timing of surgery and the extension of bone invasion also need to be considered. The surgeon may adopt a wider margin for the cases with more extensive bone invasion or longer waiting time between the imaging and surgery. The Bland–Altman analysis showed that to account for 95% of the variance in the discrepancy between margin distances measured in CT scan and pathology specimen, a leeway distance of 13 mm in general, 15 mm for malignancy cases, and 9 mm for benign cases should be considered when designing the location of osteotomy plane during VSP. For example, when the resection for a squamous cell carcinoma with bone involvement is planned, considering the definition of a clear resection margin of 5 mm by NCCN, a margin of 20 mm on the CT scan is recommended^20^.

Of note, our result alarmed surgeons that special attention shall be paid to osteosarcoma during VSP because CT bone margin distance might not reliably predict the final pathological margin distance. There was only one case with a pathologically positive margin in our series, which was radiation induced chondroblastic osteosarcoma of the mandible. We used both CT and PETCT for margin determination during VSP and the designed margin distance was 10.8 mm, but the final pathology showed one positive margin at the mandibulectomy cutting surface. Review of the histology of the tumor showed that besides the large nodules of malignant bone and cartilage in this chondroblastic osteosarcoma, there was also an infiltrative component in the marrow that invaded between the marrow fat cells, without inflammation or desmoplastic reactions. This infiltrative component could not be detected in all modalities of radiological imaging that were done prior to surgery on this patient. The determination of accurate margins in sarcoma has always been a challenge. Previous studies showed that the systematic error between the margins determined via preoperative imaging and histopathology was up to 19 mm^7^. Due to the anatomical restrictions in the head and neck area, it is often difficult to incorporate a big leeway for the possible error in resection. Patient-specific cutting guides helped reduce the error in resection. However, the determination of the margin during the design of patient-specific cutting guides remains very challenging in osteosarcoma cases.

For maxillary tumors, bony margins measured from CT were not predictable of the final pathology margins according to the result. This could be due to the smaller sample size or the more complex anatomy of maxilla such as the maxillary sinus, nasal cavity, and orbital floor, making it more difficult to assess the margin in imaging and in final pathology. The complicated anatomy of the maxilla also often warrants more complex osteotomy planes compared to the mandible. Orientation of maxillary specimen can be challenging when the normal anatomy is distorted with the tumorous growth. Maxilla was also proved to have less cortical bone than mandible, allowing more aggressive tumor growth^21^. Considering the difficulty in margin assessment, a more aggressive resection plan, providing more generous predicted margins, might be indicated in these maxilla surgeries. However, care should be taken not to overinterpret this result due to the relatively small number of maxilla cases in our study.

In the current study, a stronger correlation between the margin distances measured from CT and histopathology was detected for malignant cases than benign cases. This surprising result might be due to the difference in timing of surgeries. With priorities given to malignancy cases most of the time, both a much longer waiting time and a larger variation were recorded for benign cases (18.8±7.0 days for malignancy cases vs. 68.1±23.5 days for benign cases). This large variation in the time from imaging to operation might have led to a weaker correlation between the two measurements in benign cases.

For malignant tumors, the soft tissue margin distance was significantly smaller than the bone margin distance. This might be due to the shrinkage of the soft tissue margin after it was removed from the patient and upon fixation in routine histopathology processing, but bony tissue was not affected^4^. Besides the effect of tissue shrinkage, the publication by Singh *et al*. also proved that mucosal disease extended beyond the bone involvement area in 70.4% of the SCC cases^3^. According to the current study, soft tissue margin distance is not correlated to bone margin distance. Luckily, it is often easier to adjust the soft tissue margins according to tumor extension during the operation as the bony reconstruction plan is often not affected. When oncological safety of bony resection is guaranteed with careful VSP margin assessment, a more liberal approach shall be made to ensure adequate soft tissue margins during the surgery. Other protocols to assist soft tissue margin consideration is mandatory in the VSP of jaw tumor resection.

This study has its own limitations. It was difficult to assess some of the bony margins in the final pathology due to the difficulty in slicing the specimen. The anatomical relationship of the tumor and the adjacent structures, especially the bony parts, might be distorted during the segmentation and slicing process, making an accurate assessment of the bony margin distance difficult. Since patients with preoperative adjuvant therapy were excluded from this study, the conclusion can only be applied to the treatment-naive tumors indicated for VSP. The current study focused on the reliability of margin determination during virtual surgical surgery. No survival data was involved in the present analysis.

## Conclusion

In conclusion, this prospective study proved that bone margin distance in VSP could be considered as a surrogate outcome of pathological bone margin distance. A leeway distance of 15 mm and 9 mm during VSP should be considered for malignancy and benign cases, respectively. Extra attention needs to be paid to maxillary tumors and sarcoma cases during VSP. This finding further pushed forward the frontier in precision tumor surgery, which maximizes the preservation of function without compromising oncological safety.

## Ethical approval

This study was approved by the Institutional Review Board of the University of Hong Kong/Hospital Authority Hong Kong West Cluster (UW 15-315).

## Consent

Written informed consent was obtained from the patient for publication and any accompanying images. A copy of the written consent is available for review by the Editor-in-Chief of this journal on request.

## Sources of funding

The study was supported by Health and Medical Research Fund (Project no.: 08192096), Health Bureau, Hong Kong; General Research Fund (No. 17114722), Research Grants Council, Hong Kong; and Platform Technology Fund 2021, University Research Committee, Hong Kong. Health and Medical Research Fund, General Research Fund, and University Research Committee Platform Technology Fund of Hong Kong provide funding for research purpose. No commercial interest was involved.

## Author contribution

J.J.P.: conceptualization, data curation, formal analysis, funding acquisition, investigation, methodology, project administration, writing – original draft; A.W.I.L.: data curation, investigation, writing –review and editing; M.C.M.W.: data curation, formal analysis, writing – review and editing; W.-S.C.: data curation, project administration; writing – review and editing; G.H.: data curation, investigation, project administration, writing – review and editing; W.-F.Y.: conceptualization, writing – review and editing; Y.-X.S.: conceptualization, funding acquisition, methodology, project administration, supervision, validation, writing – review and editing.

## Conflicts of interest disclosure

The authors declare that they have no known competing financial interests or personal relationships that could have appeared to influence the work reported in this paper.

## Research registration unique identifying number (UIN)

Research Registry Unique identifying number: researchregistry9053 https://www.researchregistry.com/browse-the-registry#home/.

## Guarantor

Professor Su. E-mail: Yuxiong richsu@hku.hk.

## Data availability statement

The datasets generated and/or analyzed related to this publication are available from the corresponding author upon reasonable request.

## Provenance and peer review

Not commissioned, externally peer-reviewed.
